# Biological Response of *Lallemantia iberica* to Brassinolide Treatment under Different Watering Conditions

**DOI:** 10.3390/plants10030496

**Published:** 2021-03-05

**Authors:** Saeid Naservafaei, Yousef Sohrabi, Parviz Moradi, Eileen Mac Sweeney, Andrea Mastinu

**Affiliations:** 1 Department of Agronomy and Plant Breeding, Faculty of Agriculture, University of Kurdistan, Sanandaj 66314, Iran; s.nvafaei@agri.uok.ac.ir; 2 Research of Agricultural and Natural Resources Research Center, Zanjan 45617, Iran; p_moradi@areeo.ac.ir; 3 Department of Molecular and Translational Medicine, University of Brescia, 25123 Brescia, Italy; e.macsweeney@studenti.unibs.it

**Keywords:** *Lallemantia iberica*, antioxidant enzymes, membrane peroxidation, osmoprotectant, plant growth regulator, water limitation

## Abstract

*Lallemantia iberica* (*L. iberica*) is an important dry season medicinal plant. Drought, an important abiotic stress, adversely affects the plant’s metabolism, which can be alleviated by plant growth regulators like brassinolides. A two-year field experiment was conducted in 2017–2018 to determine the effects of three different irrigation regimes and four brassinolide concentrations on the *L. iberica* biochemical properties. A split-plot based on a completely randomized block design in three replicates was used as an experimental design with the following irrigation regimes: full watering, watering until flowering and watering until branching. These were the main plots, and 0, 0.5, 1 and 1.5 μM brassinolide concentrations were applied as the subplots. The results showed that many antioxidant enzymes and some biochemical parameters were affected by brassinolide treatment. Furthermore, the highest membrane stability and grain yield were produced in full watering treatment in the second year, and these treatments were not affected by brassinolide application. Several concentrations of brassinolide differently affected the studied treatments, and our study suggests that the amelioration of the effects of the drought stress on *L. iberica* could possibly be achieved through brassinolide-induced elevation of reactive oxygen species (ROS) scavenging defense systems. There is a need for complementary research to prove the effectiveness of foliar application of this growth regulator to improve the growth and yield of *L. iberica* under water shortage conditions.

## 1. Introduction

**Figure 1:**
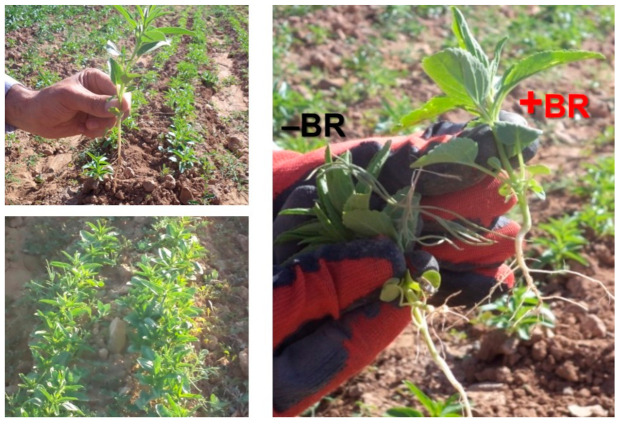
*Lallemantia iberica* crops from the Kheyr Abad site of the Agriculture and Natural Resources Research and Education Center of Zanjan (Iran). On the right, an example is shown of plant phenotype pictures with brassinolide treatment (+BR) or without brassinolide treatment (−BR).

*Lallemantia iberica* (*L. iberica*) (Figure 1), which belongs to the Lamiaceae family [1], is a source of secondary metabolites, and its seeds contain mucilage, polysaccharide, fiber, oil and protein [2]. *L. iberica* grows well in dry regions [1], and water shortage is one of the main problems for plants grown in these areas [3,4,5]. Drought is an important abiotic environmental stressor of plants [6] which often causes molecular, biochemical and physiological modifications that affect plant metabolism [7]. It has been proven that stressful conditions, along with the production of reactive oxygen species (ROS) such as superoxide anion (O_2_^−^), hydrogen peroxide (H_2_O_2_) and other free radicals, are harmful to plants and cause cell damage and cell death [8].

Antioxidant products like ascorbate, α-tocopherol and carotenoids, as well as antioxidant enzymes like catalase (CAT), ascorbate peroxidase (APX), superoxide dismutase (SOD) and glutathione reductase exist in cell organelles and the cytoplasm. They play a critical role in the detoxification of ROS [9]. It was reported that the application of certain plant growth regulators (PGRs) can improve the tolerance of different abiotic stresses like drought, heavy metal stress and salt stress [10,11,12]. Foliar application of gibberellins, citokinins, abscisic acid, brassinosteroids, salicylic acid, polyamines and proline as plant hormones and osmoprotectant have proven valuable to ameliorate stress impacts, with improved osmotic adjustment to maintain turgor and antioxidant accumulation to detoxify ROS and maintain the stability of membrane structures, enzymes and other macromolecules under drought stress [13]. Brassinolide (BR), a phytohormone belonging to the brassinosteroids family, is involved in different plant physiological processes, including adaptation to various abiotic and biotic stresses [14]. BR leads to stress tolerance by improving the plant’s defense system, which includes enhancing the activities of antioxidant enzymes (APX, SOD, peroxidase (POD), CAT and GR) [15]. Elevated activities of CAT, APX and SOD through the application of BR reduce the H_2_O_2_ content [13] and the peroxidation of membrane lipids, which is assessed by the levels of malondialdehyde (MDA) content [16].

Due to the negative effects of drought stress on herbs, a two-year field experiment was performed to investigate the effect of drought stress intensity on the membrane stability and yield of *L. iberica*. This experiment was also conducted to determine whether BR application could alleviate the degradation effects of drought stress on this plant and which concentration of BR had the best healing effect.

## 2. Results

The results of the ANOVA table showed that different concentrations of BR, as well as interaction of the year and irrigation regime, significantly affected the enzyme activity of APX (Table 1). The year and irrigation regime had significant effects on the MDA content and membrane stability. In addition, the interaction of the year, irrigation regime and BR had significant effects on the CAT, POD, SOD and PPO enzyme activity, as well as the H_2_O_2_ and proline content (Table 1).

### 2.1. Enzymes Activity

The highest activity of catalase (7.34 unit mg protein^−1^ min^−1^) was measured for year one and for the watering until branching treatment (sprayed with the solution containing 1.5 μM BR) (Table 2). The lowest activity of catalase (1.47 unit mg protein^−1^ min^−1^) was observed for year one and for full watering treatment (not treated with BR) (Table 2). In almost all irrigation regimes, BR application increased the catalase activity in *L. iberica*. For the second year, in all watering regimes, the catalase activity was augmented by increasing the BR from 0 to 1 μM, and then it decreased. Over the two years of experimentation, it was observed that the activity of catalase in plants under irrigation cut-off conditions was higher for the first year compared with the second year.

**Figure 2:**
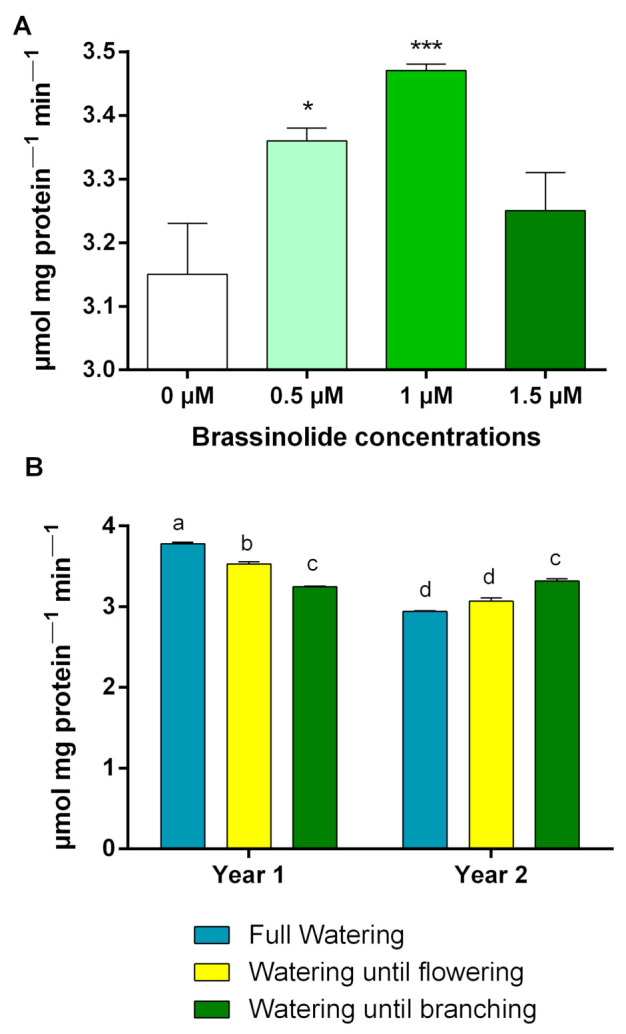
Effects of (**A**) different concentrations of brassinolides and (**B**) irrigation regimes in each year on ascorbate proxidase. According to Duncan’s multiple ranges test, bars with the same letter are not significantly different at a 5% statistical level. * *p* < 0.05 and *** *p* < 0.001 vs. control (Brassinolide 0 μM).

In year one, the activity of APX decreased with increases in the severity of water limitation, while in the second year, APX activity tended to increase (Figure 2B). The highest (3.78 μmol min^−1^) and the lowest (2.94 μmol min^−1^) amount of APX activity was observed in full watering-treated plants in year one and year two, respectively (Figure 2B). Application of 1 μM of BR also led to the highest activity of APX, which was significantly different only with the control treatment. Other treatments were placed in the same statistical group (Figure 2A).

The highest activity of peroxidase (7.32 unit mg protein^−1^ min^−1^) was obtained in year one in the watering until branching treatment (sprayed with the solution containing 0.5 μM brassinolide) (Table 2). The lowest activity of peroxidase (1.88 unit mg protein^−1^ min^−1^) was observed in year two in the full watering treatment (treated with 1.5 μM of brassinolide solution) (Table 2). In year two for all watering regimes, the activity of peroxidase was decreased by the application of increasing concentrations of brassinolide, while in year one, only the full watering regime exhibited such a trend. The results indicated that the activity of the POD enzyme in *L. iberica* plants for all irrigation regimes was higher in the first year compared with the second year (Table 2).

Like peroxidase activity, the highest activity of SOD (4.17 unit mg protein^−1^ min^−1^) was observed in year one in the watering until branching treatment (sprayed with the solution containing 0.5 μM brassinolide) (Table 2). The lowest activity of SOD (0.69 unit mg protein^−1^ min^−1^) was observed in year two in the watering until branching treatment (sprayed with 1.5 μM of brassinolide) (Table 2). In year two, all watering regimes showed a decrease in SOD activity with increasing concentrations of brassinolide (from 0 to 1.5 μM), while in year one, this trend was not observed (Table 2). The SOD activity in plants grown in the first year was significantly higher than those in the second year for all irrigation regimes and all regimes of application or non-application of brassinolide.

The results in year one showed that the PPO enzyme activity was highest (2.13 μmol min^−1^) in the watering until branching treatment regime (sprayed with 0.5 μM brassinolide) (Table 2). Its lowest activity (0.273 μmol min^−1^) was also observed in year two with the full watering treatment (treated with 1.5 μM brassinolide solution) (Table 2). The PPO enzyme activity for all irrigation treatments and all brassinolide concentration applications in the first year was higher than that in the second year.

### 2.2. Lipids Peroxidation and H_2_O_2_ Content

**Figure 3:**
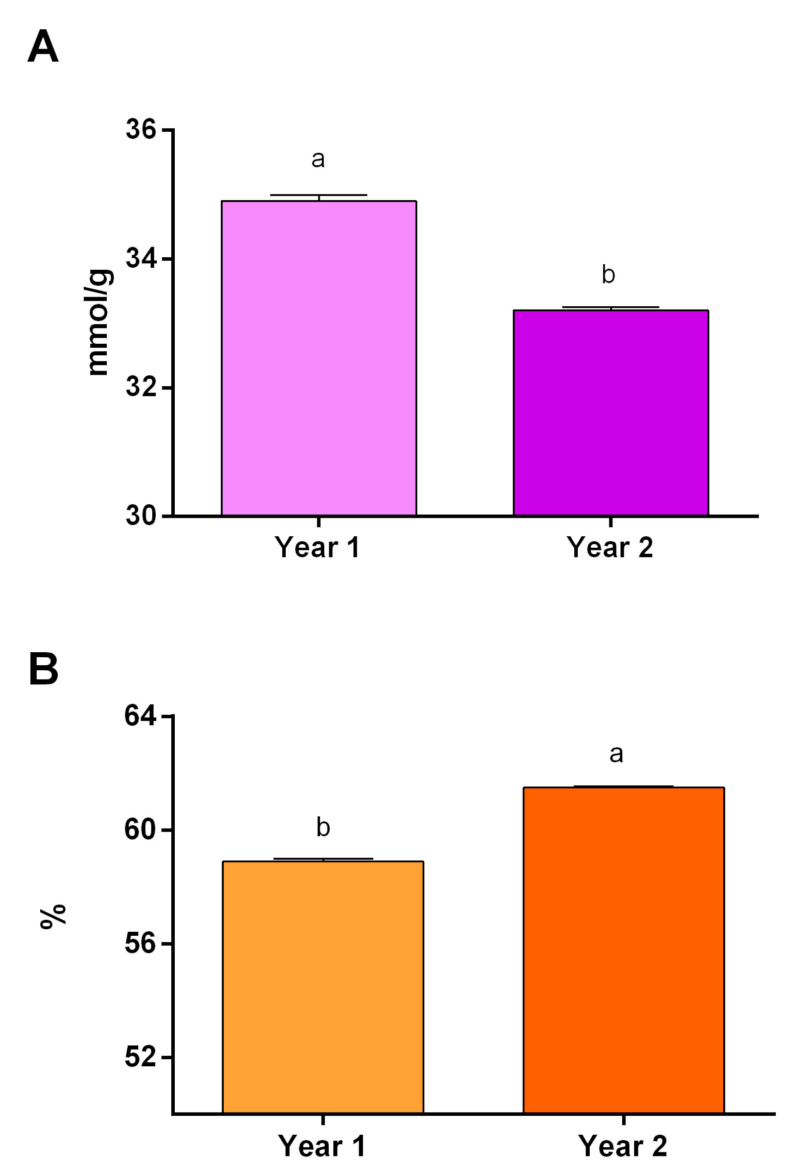
Effects of each year on (**A**) malonedialdehyde and (**B**) memberane stability. According to Duncan’s multiple ranges test, bars with the same letter are not significantly different at a 5% statistical level.

**Figure 4:**
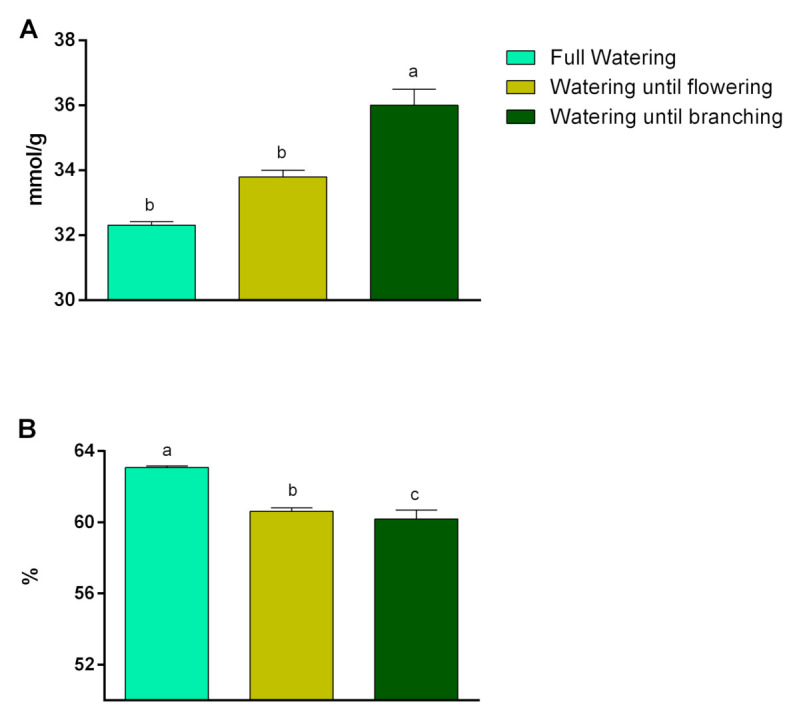
Effect of irrigation regimes on (**A**) malonedialdehyde and (**B**) memberane stability. According to Duncan’s multiple ranges test, bars with the same letter are not significantly different at a 5% statistical level.

The highest level of MDA (34.9 mmol g^−1^ FW) was produced in year one (Figure 3A). The watering until branching regime produced the highest level of MDA (36 mmol g^−1^ FW), while the full watering treatment produced the lowest level of MDA (32.31 mmol g^−1^ FW). The plants under the full watering and watering until flowering treatments were in a statistical group (Figure 4A).

The highest amount of H_2_O_2_ (169.03 mmol g^−1^ FW) was recorded in year one in the watering until branching treatment (was not sprayed with brassinolide) (Table 2). The lowest H_2_O_2_ (116.03 mmol g^−1^ FW) was observed in year two in the full watering treatment (treated with 1 μM brassinolide solution) (Table 2). Both years showed that increasing the intensity of the drought stress increased H_2_O_2_ production in *L. iberica* plants. The H_2_O_2_ content in the plant was higher in the first year than in the second year for all treatments.

### 2.3. Proline Content

The highest proline content (7.37 μmol g^−1^ FWg^−1^ FW) was found in the watering until branching treatment from year one (sprayed with 0.5 μM of brassinolide solution) (Table 2). Its lowest amount (1.78 μmol g^−1^ FW) was found in the full watering treatment from year two (sprayed with any brassinolide) (Table 2). In both years and all watering regimes (except year one and watering until branching), the proline content increased with increasing brassinolide concentrations from 0 to 1 μM and decreased with any further increase in the brassinolide concentration (Table 2). In both years, increasing the drought stress intensity resulted in higher levels of proline production in *L. iberica* plants. The highest proline content was obtained in plants under watering until branching conditions. Overall, the proline content in the first year was higher than the second year, although in some cases these differences were not significant (Table 2).

### 2.4. Membrane Stability

The highest membrane stability (61.5%) was measured in year two. The difference between the first and second years was significant in terms of this characteristic (Figure 3B). The full watering regime produced the highest level of membrane stability (63.07%), while the watering until branching regime produced the lowest level of membrane stability (61.5%) (Figure 4B). There was a significant difference between the irrigation regimes.

### 2.5. Grain Yield

**Figure 5:**
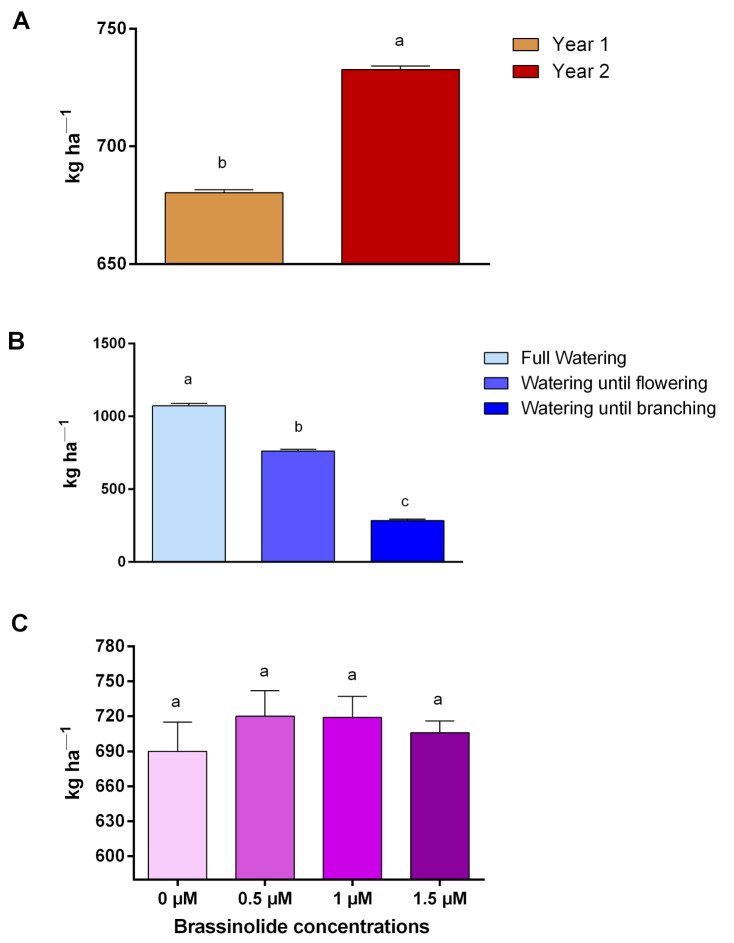
(**A**) Simple effects of the year. (**B**) Simple effects of watering regimes. (**C**) Simple effects of brassinolide concentration on grain yield of *L. iberica*.

The second year of the experiment produced the highest yield of seed (732.6 kg/h) (Figure 5A). The full watering regime produced the highest amount of seed (1073.3 kg h^−1^), while watering until branching produced the lowest amount of seed (284.3 kg h^−1^) (Figure 5B). The grain yield increased by increasing the brassinolide concentration from 0 to 1 μM, and then it decreased with further increases in concentration, although these differences were not significant (Table 1 and Figure 5C).

## 3. Discussion

### 3.1. Enzymes Activity

In both years, the watering until branching treatment produced the highest activity of catalase. In year one, its activity continuously increased by increasing the concentration of brassinolide, while in year two, this increase was observed only until the concentration of brassinolide reached 1 μM (Table 2). Supposedly, with an increase in the severity of the drought, higher concentrations of brassinolide had more inducing effects on the catalase activity, so it could be concluded that drought stress (by enhancing the H_2_O_2_ content [17]) and brassinolide cooperatively induced catalase enzyme activity. It was observed in apples that, without considering the brassinolide concentration, plants that were under more severe drought stress produced higher catalase activity [18]. Other studies have also reported that the application of brassinolide led to an increase in catalase activity [7,10].

APX is recognized as the most important peroxidase in H_2_O_2_ detoxification [19]. The application of brassinolide at 1 μM concentration had the greatest impact on APX activity (Figure 2A). The results also indicated that the lowest H_2_O_2_ content was measured at a brassinolide concentration of 1 μM. However, there was not a significant correlation between APX activity and H_2_O_2_ production (0.08, *p* ≤ 0.48) (Table 3). Observation of the opposite trend for two different years of APX activity under different intensities of stress (Figure 2B) could be a possible reason for the lack of correlation between these two traits.

For only year one of the experiment, peroxidase and superoxide dismutase activity were increased by the application of brassinolide. However, the stress intensity and applied concentration of brassinolide were the two determinant factors for the activity of this enzyme (Table 2). Positive effects from brassinolide on the peroxidase activity in drought-stressed plants were previously observed in apples [18], cowpea [15] and Indian mustard [20]. Superoxide dismutase catalyzes the reaction in which superoxide anion free radicals are detoxified and H_2_O_2_ is produced. The induction of SOD activity has been revealed to coincide with an increase in peroxidase and catalase activity [21]. Correlation analysis indicated that there was a positive and significant correlation between superoxide dismutase and peroxidase, polyphenol oxidase and catalase (Table 3). In other words, correlation analysis results indicated that in the current study, the activity of SOD, POD, CAT and PPO enzymes decreased or increased simultaneously.

Polyphenol oxidase plays an important role in controlling oxidative processes [22,23,24,25]. Although it is not considered as a component of the antioxidant defense system, its activity improved under stressful conditions [26,27]. Either water limitation or the concentration of brassinolide led to the elevation of polyphenol oxidase activity, although the effect of water limitation was more severe (Table 2). The increased activity of polyphenol oxidase under water shortage conditions could be an indicator of increased ROS generation and an accumulation of defensive mechanisms to reduce oxidative damage [28]. The results of the data mean comparison showed that the activity of catalase, peroxidase, superoxide dismutase and polyphenol oxidase in *L. iberica* plants in the first year was significantly higher than in the second year (Table 2). This result indicated that the drought’s effect was more severe in year one than year two, since the activity of these enzymes upregulated by an increase in drought stress [29].

All the antioxidant enzymes studied in this work showed their highest activity at year one rather than year two. Considering that the severity of drought stress in year one was more than year two (Table 4), as well as the positive effects of a drought on the antioxidant enzyme activity [30,31], supposedly, the severity of drought stress was one of the main reasons for the observation of higher oxidant enzymes activity for year one rather than year two.

#### Lipid Peroxidation and H_2_O_2_ Content

The highest amount of MDA was produced in the first year of the experiment (Figure 3A). In this research, like other investigations done on bananas [32], wheat [19] and chickpea [33], the lipid peroxidation significantly increased by increasing the severity of water limitation (Figure 4A), which indicated that more oxidative damage occurred [19]. According to these assertations, the obtained results and the data represented in Table 4, the severity of drought stress was greater in year one than in year two. The application of brassinolide was not effective on the MDA content (Table 1), which is in contrast to a previous study done on bananas [32]. Supposedly, an applied concentration of brassinolide could not effectively enhance the activity of the antioxidant enzymes involved in the neutralization of O_2_^−^ [7]. Therefore, the MDA content was not affected by the brassinolide application. Drought as an abiotic stress could affect plants via generating and accumulating reactive oxygen species (ROS) like H_2_O_2_, which is an inevitable by-product of normal cell metabolism [33]. Water limitation increased the H_2_O_2_ content, while the application of brassinolide ameliorated the effect of water limitation on the production of H_2_O_2_ (Table 2). This result suggests that brassinolide could possess an important role in inducing the tolerance of water limitation conditions. H_2_O_2_ can be eliminated by CAT, POD and APX [21]. According to correlation analysis, the H_2_O_2_ content significantly correlated with CAT POD, SOD and PPO (Table 3).

### 3.2. Proline

In both years, an increase in the severity of water limitation significantly enhanced the proline content (Table 2). The exogenous application of brassinolide also elevated the proline content (Table 2). Previous studies indicating that drought stress resulted in the increase of proline content in *L. iberica* [2], wheat [34], tomatoes [21] and Andean potatoes [35]. It has been suggested that the application of brassinolide upregulates the expression of proline biosynthetic genes [21]. It is proposed that proline, as a compatible solute, may assist in the drought tolerance of plants by improving osmotic adjustment, ROS detoxification, protein stabilization and cell membrane protection.

### 3.3. Cell Membrane Stability

The membrane stability in year two was higher than year one because of less rainfall, the relative humidity and increased evaporation and temperatures (drought conditions) in year one during the plant growing season (Table 4 and Figure 3B). Fully watered plants also had more membrane stability than other watering regimes (Figure 4B). Such negative effects of droughts on the membrane stability were previously observed by researchers [36,37]. Treatment with brassinolide had no significant effects on membrane stability (Table 1), which is in contrast with previous reports [15,38] which stated that brassinolide could help in maintaining the membrane integrity under drought stress.

### 3.4. Grain Yield

The lowest grain yield was obtained in year one, due to the severity of the drought stress (Figure 5A). As expected, the highest grain yield was obtained in the full watering treatment, while the lowest grain yield was obtained with the watering until branching treatment (Figure 5B). In this study, the grain yield was not affected by brassinolide application. Unfortunately, our data did not allow us to confirm the action of brassinolide that was previously observed in rice [14].

## 4. Materials and Methods

### 4.1. Plant Materials and Experimental Conditions

A two-year field experiment (2017–2018) was performed to investigate the effects of three irrigation regimes and four levels of BR treatments on the physiological and biochemical properties of *L. iberica* (Figure 1). The experiment was conducted in the Kheyr Abad site of the Agriculture and Natural Resources Research and Education Center of Zanjan, located at the northern latitude of 36° and 31′ and the eastern latitude of 48° and 45′, 1764 m above sea level. The annual average precipitation was 307 mm. Table 4 indicates the climatic properties of the field area over two years. The physical and chemical properties of the soil were analyzed using the standard methods [39] (Table 5). A split-plot on the basis of a completely randomized block design with three replicates was used as an experimental design, in which the irrigation regime, including full watering (normal or control irrigation meaning irrigation from sowing seeds (April) to the end of maturity), watering until flowering (irrigation cut-off from the beginning of flowering to the end of maturity) and watering until branching (irrigation cut-off from the emergence of the sixth pair of main stem leaves to physiological maturity), were considered as the main plots, and BR concentrations including 0, 0.5, 1, and 1.5 μM were considered as the subplots. BR was applied two times: at the six leaves stage (emergence of branches) (two L for each treatment) and at the flower initiation stage (four L for each treatment). To ensure the complete effectiveness of spraying, the treatment was done in the evening and in windless weather using a handy sprayer (FST-20DS, Fusite Co., China). The seeds of *L. iberica* were planted at a depth of 2 cm, 10 cm from one to the other and in 8 rows (25 cm apart). Plants were watered weekly until drought stress application. For the first year, irrigation cut-off occurred at the branching and flowering stages on 19 May 2017 and on 2 June 2017, respectively. For the second year, irrigation cut-off occurred at the branching and flowering stages on 23 May 2017 and on 6 June 2017, respectively. BR treatment always occurred in the afternoon and was applied with a back sprayer. Two weeks after the onset of the flowering stage, 10 plants were randomly selected, and the desired traits were measured after removing the marginal effects. The plots were 50 cm from one to the other, and there was 1.5 m between repeats. Two marginal rows, as well as 50 cm from the beginning and end of the plots, were considered as a margin.

### 4.2. Enzyme Assays

Fully expanded leaves from the middle of the main stem were sampled, immersed in liquid nitrogen and transferred to a lab. They were stored at −80 °C until enzyme extraction. Protein extraction was carried out with 500 mg of the leaf samples. The protein content was determined using the Bradford method and bovine serum albumin V (BSA V) was used as a standard [40].

#### 4.2.1. Catalase

Catalase (CAT, EC 1.11.1.6) activity was measured using the modified Beer and Sizer method as described by Sohrabi et al. [33]. The enzymatic reaction was initiated by the addition of the protein extract. The reaction mixture contained a 100 mmol L^−1^ phosphate buffer (pH 7.0), 0.1 mmol L^−1^ EDTA, 20 mmol L^−1^ H_2_O_2_ and 20 μL of protein extract. After 1 min of reaction, a spectrophotometer at 240 nm monitored the decrease of H_2_O_2_ content. H_2_O_2_ was quantified using its molar extinction coefficient (36 mol L^−1^cm). The results were expressed in CAT unit mg protein^−1^ min^−1^.

#### 4.2.2. Ascorbate Peroxidase

Ascorbate peroxidase activity (APX, EC 1.11.1.1) was determined using the method previously discussed [41]. The reaction mixture consisted of a 50 mM potassium phosphate buffer (pH 7.0), 0.5 mM ascorbic acid, 0.1 mM EDTA, 0.15 mM H_2_O_2_ and 50 µL protein extract. After 1 min of reaction, a spectrophotometer at 290 nm monitored the reduction in oxidation of ascorbic acid.

#### 4.2.3. Peroxidase

Peroxidase (POD, EC 1.11.1.7) activity was measured using the method described by Hemeda and Klein [42]. The reaction mixture contained a 25 mmol L^−1^ phosphate buffer (pH 7.0), 0.05% guaiacol, 10 mmol L^−1^ H_2_O_2_ and protein extract. The reaction was started by the addition of 60 µL of protein extract at 25 °C. After 1 min of reaction, a spectrophotometer at 470 nm monitored the increase in guaiacol oxidation, and the results were expressed as POD units per min and mg of protein.

#### 4.2.4. Polyphenol Oxidase

The Mohammadi [43] method was used to determine the polyphenol oxidase (PPO) (EC 1.10.3.1) activity. The reaction mixture consisted of 2 mL of a 0.1 M phosphate buffer (pH 6.0), 1 mL of 0.1 M catechol and 0.5 mL of protein extract. The reaction mixture was incubated for 5 min at 25 °C, and the reaction was then terminated by adding 1 mL of 2.5 N H_2_SO_4_. The increase in purpurogallin content was spectrophotometrically monitored at 495 nm. The addition of 2.5 N H_2_SO_4_ at zero time for the same assay mixture was used as the blank reaction. PPO activity was expressed in U/mg protein (U = change in 0.1 absorbance min^−1^ mg^−1^ protein).

#### 4.2.5. Superoxide Dismutase

The activity of superoxide dismutase (SOD, EC 1.15.1.1) was determined using the method previously discussed [41]. The SOD reaction mixture consisted of 50 µM ρ-nitro blue tetrazolium chloride (NBT), 1.3 µM riboflavin, 13 mM methionine, 75 nM EDTA, a 50 mM phosphate buffer (pH 7.8) and 50 µL of protein extract. The reaction mixture was irradiated under light at 78 µmol m^−2^ s^−1^ for 15 min, and its absorbance was determined spectrophotometrically at 560 nm.

### 4.3. Lipid Peroxidation and H_2_O_2_ Content

Lipid peroxidation was determined by measuring the malondialdehyde (MDA, a product of unsaturated fatty acid peroxidation) content [19]. Leaf samples (100 mg) were homogenized with 1 mL of trichloroacetic acid (20%, *w/v*) and centrifuged at 15,000× *g* for 10 min at 4 °C. An equal volume of supernatant and thiobarbituric acid (TBA) (5%) were added to the TCA (20%). The mixture was heated at 96 °C for 30 min and then placed in an ice bath for 5 min. The absorbance at 532 nm (main absorbance) and 600 nm (for correction of non-specific turbidity by subtracting the absorbance) were recorded, and the MDA content was determined using the following formula:

MDA nmol g^−1^ FM = ((A532 − A600) × V × 1000/E) × W (1)

where E is the specific extinction coefficient (155 mM cm^−1^), V is the volume of the crushed medium, W is the fresh weight of the leaf, A600 is the absorbance at 600 nm and A532 is the absorbance at 532 nm.

To determine the H_2_O_2_ content, 500 mg of fresh leaf was homogenized in 5 mL of 0.1% (*w/v*) TCA. The homogenate was centrifuged at 12,000× *g* for 20 min at 4 °C. The next step involved adding 0.5 mL of the supernatant to a 0.5 mL potassium phosphate buffer (pH 7.0, 10 mM) and 1 mL KI (1M). The absorbance of the supernatant was recorded at 390 nm. The H_2_O_2_ content was obtained using its standard curve [8].

### 4.4. Proline Content

The proline content of the leaves was determined using the method described by Bates et al. [44]. In brief, 500 mg of fresh leaf was homogenized in 10 mL of sulphosalycylic acid (3%). After centrifugation at 15,000× *g* for 10 min at 4 °C, 2 mL of the extract was transferred to a new tube, and 2 mL of glacial acetic acid and 2 mL of ninhydrin reagent were added. The tubes were boiled in a water bath at 100 °C for 30 min and then placed in an ice bath for 10 min. After cooling the reaction mixture, 6 mL of toluene was added and thoroughly mixed. The absorbance was recorded at 520 nm (toluene was used as the blank). The amount of proline (μmol/g FW) was determined using regression equations and the standard curves.

### 4.5. Membrane Stability

For the estimation of cell membrane stability, electrolyte leakage from the leaf cells was measured [45]. The method described by Ben Hamed et al. [46] was used to determine the electrolyte leakage. The first fully expanded leaves on the middle part of the main stem (two weeks after onset of the flowering stage) were excised and cut into 1 cm segments and then placed in the test tubes containing 10 mL of double-disuntiled water. The tubes were incubated in a water bath at 32 °C for 2 h, and the initial electrical conductivity of the samples (EC_1_) was measured by an EC meter (Metrohm, Filderstadt, Germany). The leaves were then sterilized in an autoclave at 121 °C for 20 min to release all the electrolytes—equilibrated to room temperature—and then the final conductivity (EC_2_) was measured. The electrolyte leakage (EL) was calculated using the following formula: EL = (EC_1_/EC_2_) × 100.4.6. Grain Yield

All the plants from the 2 m^2^ in the middle of the plot were harvested when they were ripe and dry, and the grain yield was measured.

### 4.6. Statistical Analysis

Before the data were analyzed, a normality test was performed using the Mini Tab software. A separate analysis of data variance for each year was performed on a split-plot in a randomized complete block design. Then, an error variance homogeneity test was performed for all studied traits over a two-year period using the Hartley F test. Considering that the variance of experimental errors over the two-year period was uniform for all studied traits, a combined analysis of variance of data over the two-year period was used. After ensuring the normality of the data, SAS 9.1 statistical software was used for analysis of the data variance and mean comparison. The mean of the studied traits was compared using Duncan’s test at the level of 5% probability. The Pearson correlation coefficient was also calculated for all parameters, but only the correlations between SOD, POX, PPO, CAT, APX, H_2_O_2_ and the grain yield were reported.

## 5. Conclusions

Drought stress and the increase of its intensity increased the H_2_O_2_ content, proline and lipid peroxidation of the membrane. The results of the present work suggest that brassinolide plays an important role in the reduction of the drought stress impact by enhancing the levels of osmoprotectants (proline), activities of ROS scavenging enzymes (CAT and APX) and membrane stability. The grain yield was not affected by the concentrations of brassinolide used. Since different concentrations of brassinolide did not similarly affect the studied treatments, a definite concentration of brassinolides could not be recommended. Our study suggests that the modulation of the effects of drought stress by brassinolide application on *L. iberica* can possibly be done through the stimulation of the ROS-scavenging defense systems. There is a need for complementary research to prove the effectiveness of foliar application of this growth regulator to improve the growth and yield of *L. iberica* under water shortage conditions.

## Figures and Tables

**Table 1:** Combined analysis of variance from 2017 to 2018 for catalase, ascorbate peroxidase, peroxidase, superoxide dismutase and polyphenol oxidase enzymes activity, as well as the H_2_O_2_, malondialdehyde and proline content, membrane stability and grain yield of *L. iberica* under different irrigation regimes and different concentrations of brassinolide (BR).

S.O.V.	df	Mean Square									
		CAT	APX	POD	SOD	PPO	H_2_O_2_	MDA	PRO	MS	GY
Y	1	**	**	**	**	**	**	**	**	**	**
Y (error)	4	-	-	-	-	-	-	-	-	-	-
L.W	2	ns	ns	ns	ns	ns	*	*	**	**	**
Y × L.W.	2	**	**	**	**	**	**	ns	**	ns	ns
Error (a)	8	-	-	-	-	-	-	-	-	-	-
B	3	ns	*	ns	ns	ns	**	ns	ns	ns	ns
L.W. × B	6	ns	ns	ns	ns	ns	ns	ns	ns	ns	ns
Y×B	3	**	ns	**	**	**	ns	ns	ns	ns	ns
Y × L.W. × B	6	**	ns	**	**	**	**	ns	**	ns	ns
Error (b)	36	-	-	-	-	-	-	-	-	-	-

S.O.V. = source of variation; Y = year; L.W. = limited watering; B = brassinolide concentration; C.V. = coefficient of variation; df = degree of freedom; CAT = catalase; APX = ascorbate peroxidase; POD = peroxidase; SOD = superoxide dismutase; PPO = polyphenol oxidase; H_2_O_2_ = hydrogen peroxide content; MDA = malondialdehyde; PRO = proline; MS = membrane stability; and GY = grain yield. ^ns^ not significant; * significant at 5% probability level; and ** significant at 1% probability level.

**Table 2:** Interaction of the year with the irrigation regime and the BR concentrations with the activity of catalase, peroxidase, superoxide dismutase and polyphenol oxidase enzymes and the content of hydrogen peroxide and proline.

Year	Irrigation Regime	Brassinolides Concentration	CAT ^†^ Unit mg Protein^−1^ Min^−1^	POD Unit mg Protein^−1^ Min^−1^	SOD Unit mg Protein^−1^ Min^−1^	PPO μmol Min^−1^	H_2_O_2_ mmol g^−^^1^ FW	Pro μmol g^−^^1^ FW
Year 1	Full watering	0	1.47 ^o ‡^	4.94 ^h^	1.76 ^i^	0.947 ^fg^	125.03 ^hi^	1.84 ^o^
		0.5	3.21 ^i^	4.54 ^j^	1.99 ^h^	0.793 ^hi^	120.3 ^j^	2.89 ^l^
		1	2.72 ^jk^	3.84 ^l^	2.23 ^g^	0.667 ^ij^	120.95 ^j^	2.84 ^l^
		1.5	1.75 ^n^	3.73 ^lm^	1.83 ^i^	0.603 ^jk^	124.99 ^hi^	2.46 ^m^
	Watering until flowering	0	3.63 ^h^	5.79 ^f^	2.92 ^e^	0.86 ^gh^	154.61 ^c^	3.48 ^jk^
		0.5	3.9 ^g^	6.44 ^d^	2.87 ^e^	1.54 ^c^	152.61 ^d^	3.85 ^i^
		1	4.25 ^e^	6.19 ^e^	2.73 ^f^	1.25 ^d^	150.49 ^ef^	4.01 ^hi^
		1.5	4.12 ^ef^	5.88 ^f^	2.7 ^f^	0.993 ^fg^	153.05 ^cd^	3.98 ^hi^
	Watering until branching	0	5.48 ^d^	6.18 ^e^	3.4 ^d^	1.14 ^de^	169.03 ^a^	6.67 ^cd^
		0.5	5.87 ^c^	7.32 ^a^	4.17 ^a^	2.13 ^a^	168.17 ^a^	7.37 ^a^
		1	6.58 ^b^	7.15 ^b^	3.83 ^b^	1.74 ^b^	167.47 ^a^	7.1 ^ab^
		1.5	7.34 ^a^	6.86 ^c^	3.6 ^c^	1.57 ^c^	167.83 ^a^	6.47 ^de^
Year 2	Full watering	0	2 ^m^	3.62 ^mn^	1.58 ^jk^	0.28 ^m^	120.55 ^j^	1.78 ^o^
		0.5	2.38 ^l^	3.51 ^n^	1.33 ^l^	0.347 ^m^	117.74 ^k^	1.95 ^o^
		1	2.41 ^l^	2.87 ^o^	0.93 ^no^	0.417 ^lm^	116.04 ^l^	2.21 ^mn^
		1.5	2.35 ^l^	1.88 ^q^	0.89 ^no^	0.273 ^m^	120.6 ^j^	1.84 ^o^
	Watering until flowering	0	2.77 ^jk^	4.88 ^h^	1.52 ^k^	0.58 ^jk^	126.64 ^gh^	3.24 ^k^
		0.5	2.91 ^j^	4.73 ^i^	1.09 ^m^	0.5 ^kl^	127.21 ^g^	3.74 ^ij^
		1	2.78 ^jk^	4.26 ^k^	1 ^mno^	0.41 ^k−m^	124.62 ^i^	4.64 ^g^
		1.5	2.7 ^k^	3.79 ^l^	0.88 ^no^	0.29 ^m^	125.93 ^g−i^	4.22 ^h^
	Watering until branching	0	3.19 ^i^	5.64 ^g^	1.7 ^ij^	0.49 ^kl^	156.42 ^b^	5.8 ^f^
		0.5	3.45 ^h^	4.22 ^k^	1.03 ^mn^	0.68 ^ij^	149.33 ^f^	6.85 ^bc^
		1	4.03 ^g^	3.59 ^n^	0.94 ^no^	0.79 ^hi^	151.91 ^de^	7.26 ^a^
		1.5	3.89 ^g^	2.57 ^p^	0.69 ^p^	1.03 ^ef^	154.4 ^c^	6.33 ^e^

‡ According to Duncan’s multiple ranges test, values followed by the same letter within the columns are not significantly different at a 5% statistical level. † CAT = catalase; POD = peroxidase; SOD = superoxide dismutase; PPO = polyphenol oxidase; H_2_O_2_ = hydrogen peroxide; and Pro = proline.

**Table 3:** Correlation between antioxidant enzymes, H_2_O_2_ and grain yield.

	SOD	POD	PPO	CAT	APX	H_2_O_2_	GY
SOD	1	0.88147 **	0.83144 **	0.74785 **	0.22676 ^ns^	0.67906 **	−0.3992 ^ns^
POD	0.88147 **	1	0.78694 **	0.71968 **	0.20204 ^ns^	0.72549 **	−0.5287 **
PPO	0.83144 **	0.78694 **	1	0.80645 **	0.26597 *	0.77561 **	−0.5882 **
CAT	0.74785 **	0.71968 **	0.80645 **	1	0.04098 ^ns^	0.85996 **	−0.7761 **
APX	0.22676 ^ns^	0.20204 ^ns^	0.26597 *	0.04098 ^ns^	1	0.08424 ^ns^	0.03295 ^ns^
H_2_O_2_	0.67906 **	0.72549 **	0.77561 **	0.85996 **	0.08424 ^ns^	1	−0.8991 **
GY	−0.3990 **	−0.5287 **	−0.5882 **	−0.7761 **	0.03295 ^ns^	−0.8991 **	1

CAT = catalase; APX = ascorbate peroxidase; POD = peroxidase; SOD = superoxide dismutase; PPO = polyphenol oxidase; H_2_O_2_ = hydrogen peroxide content; MDA = malondialdehyde; and GY = grain yield. ^ns^ Not significant. * Significant at a 5% probability level. ** Significant at a 1% probability level.

**Table 4:** Climatic data of the experimental site during the *L. iberica* growing season.

	April		May		June		July		August	
	2017	2018	2017	2018	2017	2018	2017	2018	2017	2018
Precipitation (mm)	31.5	24.6	25.7	85.8	0	57	1.5	0	0.8	0
Temperature (°C)	8.3	9.8	14.4	11.8	17.8	17.7	22.5	24	23.3	24.6
Evaporation (mm)	-	-	178.7	130	264.9	204.8	324.8	311.4	318	308.9
Relative humidity (%)	59	49	52	65	30	55	32	33	33	40

**Table 5:** The physical and chemical properties of the experimental site soil.

	Depth	Texture	Nitrogen Total (%)	Absorbable Phosphorus (ppm)	Absorbable Potassium (ppm)	EC (mmhos/cm)	pH
2017	0–30	Loam/Clay	0.51	12.1	463	3.13	7.4
2018	0–30	Loam/Clay	0.49	11.8	435	3.51	7.1

## Data Availability

The data presented in this study are available on request from the corresponding author.
